# metabCombiner 2.0: Disparate Multi-Dataset Feature Alignment for LC-MS Metabolomics

**DOI:** 10.3390/metabo14020125

**Published:** 2024-02-15

**Authors:** Hani Habra, Jennifer L. Meijer, Tong Shen, Oliver Fiehn, David A. Gaul, Facundo M. Fernández, Kaitlin R. Rempfert, Thomas O. Metz, Karen E. Peterson, Charles R. Evans, Alla Karnovsky

**Affiliations:** 1 Department of Computational Medicine and Bioinformatics, University of Michigan Medical School, Ann Arbor, MI 48109, USA; akarnovs@med.umich.edu; 2 Department of Medicine, Geisel School of Medicine, Dartmouth College, Hanover, NH 03755, USA; jennifer.l.meijer@hitchcock.org; 3 West Coast Metabolomics Center, University of California, Davis, CA 95616, USA; tsshen@ucdavis.edu (T.S.); ofiehn@ucdavis.edu (O.F.); 4 School of Chemistry and Biochemistry, Georgia Institute of Technology, 901 Atlantic Drive, Atlanta, GA 30332, USA; david.gaul@chemistry.gatech.edu (D.A.G.); facundo.fernandez@chemistry.gatech.edu (F.M.F.); 5 Biological Sciences Division, Pacific Northwest National Laboratory, Richland, WA 99352, USA; kaitlin.rempfert@pnnl.gov (K.R.R.); thomas.metz@pnnl.gov (T.O.M.); 6 Department of Nutritional Sciences, University of Michigan School of Public Health, Ann Arbor, MI 48109, USA; karenep@umich.edu; 7 Department of Environmental Health Sciences, University of Michigan School of Public Health, Ann Arbor, MI 48109, USA; 8 Department of Internal Medicine, University of Michigan Medical School, Ann Arbor, MI 48109, USA; chevans@med.umich.edu

**Keywords:** metabolomics, LC-MS, alignment, chromatography, R package, software

## Abstract

Liquid chromatography–high-resolution mass spectrometry (LC-HRMS), as applied to untargeted metabolomics, enables the simultaneous detection of thousands of small molecules, generating complex datasets. Alignment is a crucial step in data processing pipelines, whereby LC-MS features derived from common ions are assembled into a unified matrix amenable to further analysis. Variability in the analytical factors that influence liquid chromatography separations complicates data alignment. This is prominent when aligning data acquired in different laboratories, generated using non-identical instruments, or between batches from large-scale studies. Previously, we developed metabCombiner for aligning disparately acquired LC-MS metabolomics datasets. Here, we report significant upgrades to metabCombiner that enable the stepwise alignment of multiple untargeted LC-MS metabolomics datasets, facilitating inter-laboratory reproducibility studies. To accomplish this, a “primary” feature list is used as a template for matching compounds in “target” feature lists. We demonstrate this workflow by aligning four lipidomics datasets from core laboratories generated using each institution’s in-house LC-MS instrumentation and methods. We also introduce batchCombine, an application of the metabCombiner framework for aligning experiments composed of multiple batches. metabCombiner is available as an R package on Github and Bioconductor, along with a new online version implemented as an R Shiny App.

## 1. Introduction

Metabolomics is widely used in biological research to assess the small molecule composition of biological samples and derive insights into physiological phenomena. The leading technology for untargeted metabolomics is liquid chromatography–mass spectrometry (LC-MS), which routinely detects thousands of metabolomics features with high sensitivity and measures their mass-to-charge ratio (m/z), retention time (RT), and relative abundance [1]. Feature alignment across samples is a key step in computational metabolomics workflows; many published software methods [2] correct for chromatographic drift due to changes in temperature, column age, sample instability, and solvent proportioning [3,4,5]. Large-scale metabolomics studies composed of multiple batches frequently harbor significant drift in RTs within, and especially between, batches [6,7,8]. LC-MS metabolomics data obtained with non-identical instruments and parameters often display greater RT differences (up to several min) that cannot be overcome using conventional alignment approaches. As efforts to standardize protocols have not yet substantially materialized in the metabolomics field, there are significant challenges to data interoperability within and between laboratories. Only a few strategies have been devised for disparate LC-MS method metabolomics feature alignment [9,10,11].

Our group has previously described metabCombiner, a software package for aligning experimentally incongruent LC-MS metabolomics datasets [12]. metabCombiner bridges wide differences in analytical parameters, such as chromatographic gradients, column dimensions, and mass spectrometer settings, to construct a merged feature matrix. metabCombiner was initially designed for the alignment of two pre-processed feature tables, with limited functionality for handling three or more table inputs. The program reported all hypothetical paired alignments but lacked approaches for the automated assignment of one-to-one feature correspondence. The program reported an intersected feature list, removing compounds lacking suitable matches.

In this work, we describe major additions to metabCombiner, extending the original method to enable stepwise metabolomics data-merging operations. The updated package contains functionality for aligning combinations of feature tables, as well as automated correspondence assignment and the inclusion of non-intersected features for subsequent alignment steps. As part of this framework, we implemented a new method called batchCombine for aligning multi-batch LC-MS metabolomics experiments, which serves as an alternative to processing often unmanageably large datasets in a single batch. We illustrate these methods in two different metabolomics studies. First, we use metabCombiner 2.0 to align data from an inter-laboratory untargeted lipidomics study conducted by four core laboratories, assessing the reproducibility of lipidomics identification strategies across diverse mammalian specimens. Second, we applied batchCombine to the ELEMENT (Early Life Exposures in Mexico to Environmental Toxicants) cohort study [13], which generated multi-batch untargeted LC-MS metabolomics analyses of fasting blood serum from Mexican adolescents. We also present a graphical user interface implemented as an R Shiny App to facilitate the package’s usage by the wider metabolomics community.

## 2. Materials and Methods

### 2.1. Workflow Overview

**Figure 1:**
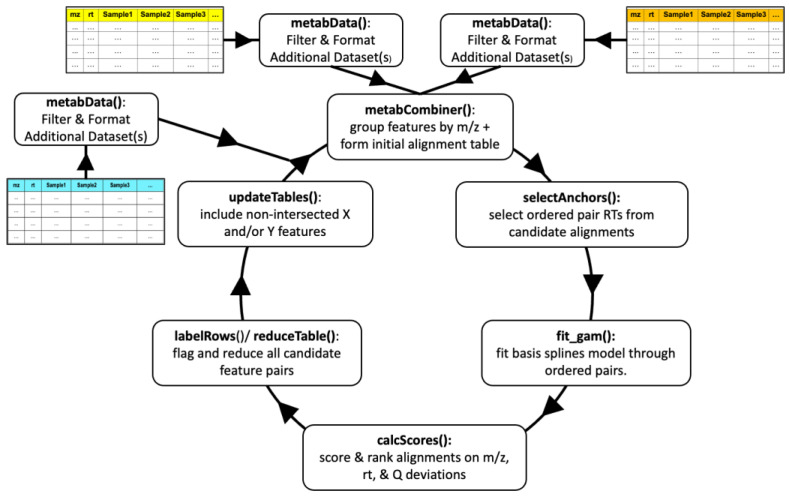
metabCombiner 2.0 workflow.

The upgraded metabCombiner workflow is depicted in Figure 1. The new alignment process is illustrated as a cycle, starting with the merging of two metabolomics feature tables, followed by the integration of additional single or combined datasets, with each cycle comprised of six alignment steps. Three of the steps, selectAnchors, fit_gam, and calcScores, perform as described previously [12] with minor changes. The metabCombiner() step, which serves to determine the set of possible feature alignments (constrained by m/z) was revamped to accommodate multi-dataset objects and the organization of data into constituent feature lists. A new function, reduceTable, was developed to enable automatic assignment of one-to-one correspondence between feature pairs, according to calculated alignment scores and ranks. Finally, a new function called updateTables restores features from X and Y dataset inputs to the results table that lack complementary paired matches. Each step is described in detail below.

### 2.2. Terminology

The package contains two objects for representing metabolomics data: metabData and metabCombiner. metabData objects are single dataset representation classes containing a formatted feature table, with each feature described by the mass-to-charge ratio (m/z), retention time (RT), and per-sample abundance values (samples). Optional identifiers (id), and adduct or formula labels (adduct) are stored for each feature as well. Any additional columns may be designated as “extra”, and are reported in the final output but do not affect the alignment. Features in metabData objects are assigned a quantile abundance value (Q) between 0 (least abundant) and 1 (most abundant), based on the ranked median or mean peak intensities across samples. metabCombiner objects are multi-dataset representation classes that serve as the main framework for executing the package workflow steps. Two closely linked report tables are generated in each alignment: a combined table containing the set of possible feature pair alignments (FPAs), their associated per-sample abundances, and alignment scores for the feature pairs; and the feature data table, which organizes the aligned features and their associated descriptors (m/z, RT, id, adduct, Q) by the constituent dataset of origin. Each dataset is assigned its own unique identifier.

### 2.3. Initial Data Processing

The inputs for metabCombiner are pre-processed LC-MS metabolomics feature tables generated using programs such as XCMS [14], MZmine [15], or MS-DIAL [16]. All feature tables must be processed and formatted as metabData objects before alignment. The associated constructor, metabData(), maps the required and auxiliary feature meta-data to input table columns and applies multiple filters to exclude features based on proportion missingness and RT ranges. Duplicate features representing the same compound can be filtered or merged into a single representative row (see Supplementary Text S1 for more details). The remaining features are eligible for alignment with other feature lists.

### 2.4. Combined Dataset Construction

**Figure 2:**
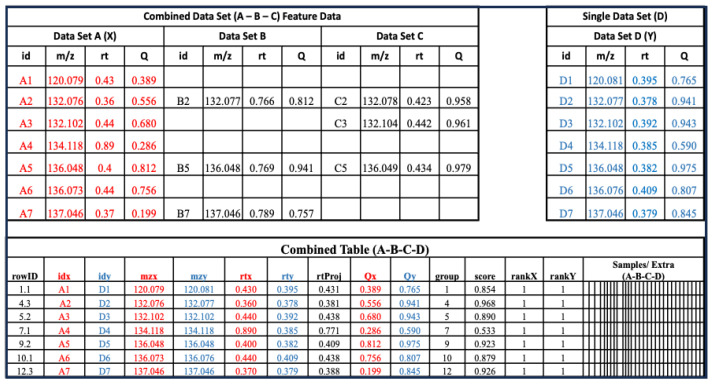
A multi-dataset object consisting of three previously aligned feature lists (A, B, and C) is aligned to a new single dataset (D), with one constituent list (Data Set A, red columns) selected to represent the combined entity for feature matching with (Data Set D, blue columns).

A metabCombiner multi-dataset class can be constructed from two single datasets, a single and a combined dataset, or two combined dataset objects. In each cycle, one input object is selected to be the projection (“X”) feature list and the other is designated as the reference (“Y”). Figure 2 illustrates a combined dataset composed of three feature lists ‘A’, ‘B’, and ‘C’, and a new single dataset labeled as ‘D’. One set of feature descriptors is selected from combined dataset (A-B-C) for comparison with new list D. In Figure 2, for example, X columns are drawn from feature list A, whereas Y columns are derived from dataset D. This generates the combined table showing the list of possible FPAs arranged into m/z-based groups, constrained by the ‘binGap’ parameter. The columns of this table are the same as described previously, including a ‘score’ column that represents the calculated similarity of the pairs; rankX and rankY ordering the alignment scores by individual features; and “rtProj”, showing the mapping of retention times from the projection set (rtx) to the reference (rty). metabCombiner also updates the feature data table to include table D features, with the two results tables linked by a single column (“rowID”).

### 2.5. RT Mapping and Score Calculation

Briefly, a subset of the feature pairs is chosen among highly abundant compounds (within m/z, RT, and Q difference constraints) for modeling the retention time warping function between the projection and reference chromatograms (selectAnchors). fit_gam() computes a penalized basis spline model to obtain RT mapping, using the selected anchors from the previous step. This mapping can be further constrained to an RT range by removing empty head or tail chromatographic regions. Subsequently, calcScores() computes a similarity score between 0 and 1 for all grouped feature pairs, using an exponential penalty for differences in m/z, RT (model-projected rt_y_ vs. observed rt_y_), and Q. Pairwise score ranks (rank_X_ and rank_Y_) are computed for each unique feature with respect to their complements. The most plausible matches are ranked first (rank_X_ = 1 and rank_Y_ = 1) and score close to 1.

### 2.6. Aligned Feature Table Annotation and Reduction

Using the computed scores and ranks, the initial combined feature table can be reduced to a final list of plausible feature matches. Simple thresholds for alignment scores, pairwise ranks, and RT prediction errors typically exclude over 90% of mismatches. To consider competitive alignment hypotheses among lower-ranked feature pairs (e.g., alignments between closely eluting isomers), the program identifies rows that (i) meet the indicated score and rank thresholds; (ii) have a feature in common with a top-ranked alignment (rank_X_ = 1, rank_Y_ = 1); and (iii) have similarity score values (or alternatively m/z and RT) within a small gap of the top-ranked alignment. FPAs that meet conditions (i–iii) are flagged as conflicting possibilities, and associated rows are assigned to a “subgroup”. On the other hand, rows that meet (i) and (ii), but not (iii) are deemed removable. To resolve conflicts, a third step was added to select the combination of FPAs within each subgroup with the highest sum of scores. Finally, all rows annotated as removable are eliminated from the tables. See Table S1 for illustrative examples of this process.

### 2.7. Assembling Aligned and Non-Aligned Features

Following the table reduction step, many input features will not be aligned due to a lack of complementary matches. updateTables() locates all features present in the original X or Y input objects used to construct the object, but missing in the reduced aligned data table. These features are carried forward into both report tables, with missing values in counterpart data columns. This allows for non-intersected features to be used in further alignment steps and serves as the final step of the package workflow. Users may opt to forgo this step if only the intersection is desired, though it is highly recommended that this step be performed when aligning more than two input feature lists.

### 2.8. batchCombine: Extension to Multi-Batch LC-MS Alignment Tasks

The updated metabCombiner framework has applications for large-scale metabolomics studies composed of multiple batches. As previously noted, instrumental conditions often undergo subtle changes throughout the course of a large-scale experiment. LC-MS run order is an important variable as distal batches tend to be more dissimilar compared to proximal sample runs [8]. For a study composed of *N* batches, the batchCombine procedure requires each batch of samples be separately pre-processed, generating *N* total feature tables. After transforming each table into metabData format and arranging in run order, the batchCombine wraps together the six alignment steps to construct a batch-merged object in a stepwise manner. The first batch is aligned to the second, and the results are then aligned to the third, and so forth, until a total of *N−1* alignment cycles are complete. In each cycle, feature descriptors are drawn from the latter batch or, alternatively, the averaged <m/z, RT, Q> values among previously merged sample sets for comparison with the next batch. batchCombine uses identical parameters in each cycle, with stricter default values for retention time modeling weights and tolerances.

### 2.9. metabCombiner Online

We have developed an R Shiny App web interface for the program, implementing the functionality of the R package. metabCombiner Online is divided into two main tabs: metabCombine for pairwise disparate LC-MS alignment and batchCombine for large multi-batch metabolomics studies. The app accepts as input .csv or .txt files for single datasets or .rds files for combined dataset objects. For the metabCombine tab (default workflow), the app generates customizable plots for the spline-fitted retention time model, as well as summary information for the input and output dataset objects. A link to the app, as well as a detailed guide to its usage and links to the R package versions on Github and Bioconductor, can be found at https://metabcombiner.medicine.umich.edu/ (accessed on 1 January 2024).

### 2.10. Unknown Lipids Consortium Study

#### 2.10.1. Study Design and Experimental Methods

We demonstrate metabCombiner on an inter-laboratory untargeted lipidomics study conducted at four laboratories (hereafter designated as “I”, “II”, “III”, and “IV”) operating as NIH Compound Identification Development Cores (CIDCs). The laboratories measured the lipid profiles of multiple mammalian specimens, namely 4 pooled human plasma samples provided by NIST and 1 commercially available lipid extract each of human muscle, bovine heart, bovine liver, and porcine brain (Avanti Polar Lipids, Alabaster, AL, USA). Each laboratory acquired data using their own individualized SOP for untargeted lipidomics. Detailed descriptions of the methods used in the study were previously reported [17], using different identifiers to the ones used here (A = III, B = IV, C = I, D = II). The instruments used by the laboratories include a Shimadzu CTO-20A Nexera X2 UHPLC coupled to an AB Sciex 5600 TripleTOF (I) (AB Sciex, Concord, ON, Canada), a Thermo Vanquish UPLC coupled to a Thermo Q-Exactive HF (II) (Thermo Fisher Scientific, San Jose, CA, USA), an Agilent 1290 Infinity II coupled to an Agilent 6530 QTOF (III) (Agilent Technologies, Inc., Santa Clara, CA USA), and a Waters—ACQUITY UPLC H-Class PLUS System (Waters Corporation, Milford, MA, USA) coupled to a Thermo LTQ-Orbitrap Velos (IV). Chromatographic columns used were Waters HSS T3 C18, 50 mm × 2.1 mm ID, 1.8 µm particle diam (I), Thermo Accucore C30, 2.1 mm × 150 mm, 2.6 μm particle column (II), Waters ACQUITY UPLC CSH C18, 2.1 mm × 100 mm, 1.7 μm particle column (III), and a Waters CSH C18 column (3.0 mm × 150 mm, 1.7 µm particle size) (IV). For plasma samples, various lipid extraction methods were used, namely, modified Bligh–Dyer (I), single-step isopropanol precipitation (II), Matyash (III), and modified Folch (IV).

#### 2.10.2. Data Processing and Metabolite Identification

Each participating laboratory performed conventional pre-processing (peak picking, alignment across samples) for their own respective dataset. They also acquired MS/MS spectra and identified as many lipids as possible using tandem MS library searches [17]. Laboratory I used MS-DIAL [16] for lipid identification and Multiquant (AB SCIEX, Concord, ON, Canada) for quantitation. Laboratory II used Compound Discoverer (Thermo Fisher Scientific, San Jose, CA, USA) and Laboratory III used MS-DIAL [16] for both identification and quantitation. Laboratory IV raw files were pre-processed with the MZmine2 [15] software with the ADAP modules [18]. The laboratories provided pre-processed and annotated data tables in various formats, with no post-processing steps performed to filter the resulting feature lists.

#### 2.10.3. Stepwise Alignment with metabCombiner

For this analysis, peak area columns corresponding to human plasma samples were selected for relative quantitation comparisons since they comprised the largest share of the LC-MS data in the study (12 total sample runs) compared to other specimen types (3 runs each). Other data columns were incorporated as ‘extra’ into the eventual aligned feature table. RT range constraints were imposed on two feature tables (I and IV) to improve modeling performance by eliminating chromatographic regions consisting mostly of non-biological features. Features with missing values in more than 50% of plasma samples were removed by default. Duplicate features (defined as two or more rows with close m/z and RT values, representing the same compound) were highly prevalent in dataset I; wherever detected, these rows were merged as described in Supplementary Text S1.

The feature tables provided by the laboratories were aligned to each other in a stepwise manner using metabCombiner, with customized parameters for each pairwise operation. First, Table 1 I and II were combined, and the resulting combined table object was aligned to III, followed by IV. In each alignment cycle, meta-data from feature list I were selected for comparison with the next list, thereby centering all alignments to list I features. metabCombiner generated the set of feature alignments, as well as a concatenated data table from these four datasets. The script for performing the stepwise alignment can be found in Supplementary Text S2. 

### 2.11. Early Life Exposures in Mexico to Eevironmental Toxicants (ELEMENT)

#### 2.11.1. Study Design and Experimental Methods

The Early Life Exposure in Mexico to Environmental Toxicants (ELEMENT) study was founded in the early 1990s as previously described [13]. The blood samples of 402 adolescent volunteers from the ELEMENT 2015 study visit were analyzed with an untargeted metabolomics assay at the Michigan Regional Comprehensive Metabolomics Resource Core (MRC^2^) [19]. Serum samples were thawed on ice prior to processing. For deproteinization, 100 µL of plasma was combined with 400 µL 1:1:1 methanol/acetone/water containing the following internal standards: (L-(D4)Thymine, L-[15N] Anthranilic acid (each 5 µmol/L); L-(15N)2 Tryptophan, Gibberellic acid, and L-Epibrassinolide (each 20 µmol/L)). Samples were vortexed and then centrifuged (10 min at 15,000× *g*). For RPLC-MS analysis, the supernatant was transferred to a clean vial and dried under nitrogen gas. The dried sample was reconstituted in 50 µL of methanol/water (50:50) containing zeatin (1 µmol/L). Samples were analyzed using an Agilent 1290 LC/6530 qTOF MS system (Agilent Technologies, Inc., Santa Clara, CA, USA) using a Waters Acquity HSS T3 2.1 × 100 mm column with 1.8 µm particle diameter (Waters Corporation, Milford, MA, USA). Mobile phase A was 100% water and mobile phase B was 100% methanol, both with 0.1% formic acid. The gradient was as follows: 0 min to 20 min 2% to 75% B (linear), 20 min to 22 min 75% to 98% B (linear), followed by a 7 min re-equilibration at starting conditions. The flow rate was 0.45 mL/min and the column temperature was 55 °C. Mass spectrometry was performed via electrospray ionization with an Agilent Jetstream ion source, with full-scan mass spectra acquired over the m/z range from 50 Da to 1500 Da in positive ion mode. Source parameters were: drying gas temperature of 350 °C, drying gas flow rate of 10 L/min, nebulizer pressure of 30 psig, sheath gas temp of 350 °C and flow of 11 L/min, capillary voltage of 3500 V, and internal reference mass correction enabled.

#### 2.11.2. Data Pre-Processing

All Agilent .d files were converted to .mzML format using the MSConvert tool in Proteowizard [20], organized into eight separate directories corresponding to each experimental batch, and pre-processed with XCMS [16]. The same parameters were used for pre-processing each of the batches. Peak picking is performed with the Centwave algorithm [21], with peak width from = 3 s to 30 s, noise = 250, ppm = 20, prefilter = c(3, 1000), snthresh = 10, integrate = 1, fitgauss = TRUE. Initial peak grouping was performed with the density method, with bw = 15, mzwid = 0.015, and minfrac = 0.5. Retention time correction follows, based using the loess method, with span = 0.25 and family = “symmetric.” A second density-based grouping of features was performed with bw = 5, mzwid = 0.015, and minfrac = 0.5. Gap filling is then applied using fillPeaks(). The peak tables were written to separate .csv files. 

#### 2.11.3. Alignment with batchCombine

The batch-wise feature tables were loaded into R and converted to a list of metabData objects. The “samples” field was chosen to correspond to pooled QC samples, with all other samples designated as “extra”, with all other metabData constructor parameters kept at their default values. A common binGap value of 0.0075 was employed for grouping features by m/z. Anchor selection and spline-fitting parameters were kept mostly to default values. Score calculation parameters were set to A = 70 (m/z weight), B = 35 (RT weight), and C = 0.8 (Q weight). Table reduction parameters were set to their defaults, except for maxRTerr = 0.3 min. All three quantitative descriptors—m/z, RT, Q—were averaged after every step (means = TRUE). The script for performing the batchCombine operation is provided in Supplementary Text S2, and the pre-processed batches are provided as an example dataset within metabCombiner. 

## 3. Results

### 3.1. Aligning Multi-Laboratory Untargeted Lipidomics with metabCombiner 2.0

#### 3.1.1. Unknown Lipids Study

Each of the four CIDC core laboratories performed untargeted LC-MS and LC-MS/MS analyses on identical extracts using independently devised “in-house” standard operating procedures (SOPs) typically performed for untargeted lipidomics at their respective laboratories. The SOPs used to generate these datasets differed in many important analytical parameters, including instrumentation, chromatographic columns and gradients, mass spectrometer settings, data acquisition parameters, and data preprocessing methods, as previously detailed [17]. The initial feature table sizes ranged from 2000 to 30,000 features, influenced by multiple factors such as lipid extraction efficiency, separation, instrument sensitivity, and data preprocessing workflows. Further processing by metabCombiner to reduce highly missing and duplicate features, as well as features in the boundary (solvent and re-equilibration) regions, yielded individual dataset feature counts of 8770 (I), 3809 (II), 24,905 (III), and 19,369 (IV) in the positive ion mode and 5863 (I), 2247 (II), 7308 (III), and 8935 (IV) in the negative ion mode. The full results for the individual data processing are displayed in Supplementary Table S2.

#### 3.1.2. m/z Grouping and RT Mapping Results

For all pairwise alignments, we employ a common binGap value of 0.0075 Da for grouping features in m/z space and determining the initial set of possible pairwise alignments. In the positive mode, 3355, 6241, and 5711 out of 8770 processed table I features were initially grouped by m/z with at least one unique feature from tables II, III, IV; in the negative mode, 1828, 3589, and 3755 out of 5863 table I features were assigned groups. The other tables’ features had similar grouping rates of 30 to 55%. Increasing the binGap value (up to 0.02) only slightly raises the number of unique grouped features in this analysis.

**Figure 3:**
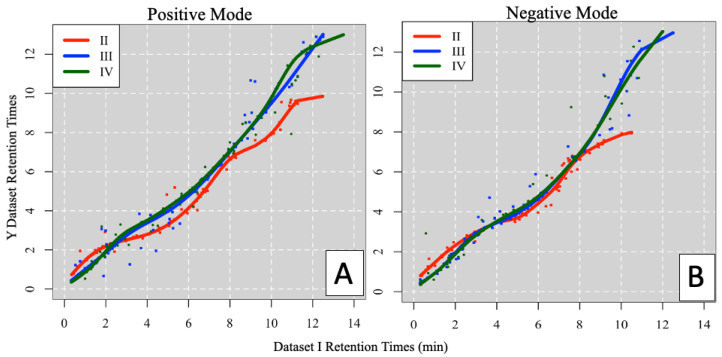
Unknown lipids inter-laboratory study RT mapping from laboratory dataset I to datasets II, III, and IV in the positive (**A**) and negative (**B**) ionization modes.

Ordered pair anchor selection and RT mapping were performed using parameters tuned to each alignment, with visual guidance from the plot method. Figure 3 illustrates the RT mapping curves for each alignment in both ionization modes, with the pairwise curves displayed in Supplementary Figure S1. Generally, the early portions of the gradient are well mapped, whereas later chromatographic regions contain poorly resolved peaks that resemble background ions. After translating RTs from data set I to each of the three counterpart datasets, similarity scoring and table reduction steps determine the most plausible one-to-one feature matches.

#### 3.1.3. Inter-Laboratory Alignment Results

Table 1 summarizes the alignment results between the four datasets. The overlaps of dataset I with sets II, III, and IV consist of 1192, 2389, and 2204 features, respectively, in the positive mode and 571, 1176, and 1533 features in the negative mode. In total, 3568 positive-mode and 2207 negative-mode list I features (roughly 40% of the post-processed feature table sizes) were aligned with at least one feature detected by the other participating laboratories. Only 601 and 215 features were aligned across all four input tables in the positive and negative ionization modes, respectively. These compounds had median Q_I_ values of 0.724 (positive) and 0.881 (negative), indicating that these features mostly represent highly abundant lipids. On the other hand, nearly 60% of processed list I features were unique to this dataset, with no matches with features in the other lists. The full aligned table reports can be found in Supplementary Sheet S1. Multiple factors could explain this relatively modest overlap. The initial sizes of pre-processed feature tables have a moderate influence, with the smallest table (II) contributing the fewest features to the overall intersection. Many absent peaks are present in the raw data but were not extracted by the pre-processing algorithms used to generate the datasets. In each pairwise alignment operation, 30 to 70% of Table I features lacked m/z-matched counterparts in the target datasets and were therefore not assigned to an m/z group. An additional 20% to 30% of dataset I features could not be aligned due to low similarity scores (<0.5) with all potential pairwise matches. Many non-aligned features derived from noise, contaminants, or in-source ion activation specific to one experiment. Finally, due to the complexity of the lipidome, which contains numerous isomeric and isobaric species, the datasets contain many peak groups within close m/z and RT distances to each other. Assigning one-to-one feature correspondence across datasets is especially challenging for isomers, particularly whenever an unequal number of peaks are detected between datasets. Previous studies have reported overlaps as small as 10% for the same dataset using different pre-processing software and parameters [22,23]. Here, different software packages were employed by separate laboratories on data acquired under non-identical conditions.

#### 3.1.4. Comparative Lipid Annotation Analysis

**Figure 4:**
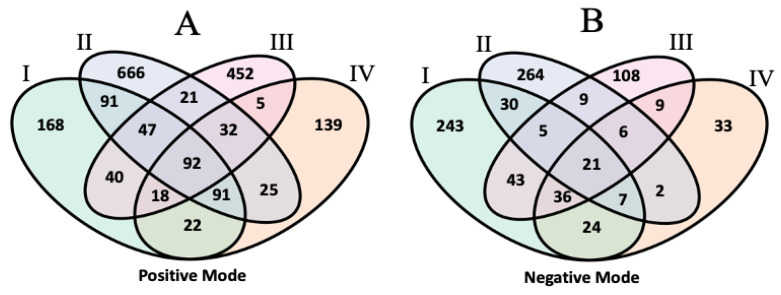
Venn diagram of the shared compound annotations in the unknown lipids study for positive (**A**) and negative (**B**) mode data.

One of the aims of the inter-laboratory lipidomics study was to compare lipid annotations assigned by the institutional participants. The overall annotation rates differed between the laboratories, with laboratory II having the highest proportion of annotated features at 20% to 30% in both ionization modes, but the lowest total number of detected features. Other laboratories reported more features but annotated fewer than 10% of them. Using the aligned feature table, we studied commonalities and differences in identity assignments for putative matched features. In this analysis, lipid annotations are treated as consistent between datasets if they match at the species level according to the shorthand nomenclature (lipid class, followed by total carbon atoms/total double bonds) proposed by the International Lipid Classification and Nomenclature Committee [24]. The results are illustrated in Figure 4.

Out of 601 positive mode features overlapped across the four lists, 92 had matching identities, including 25 phosphatidylcholines (PCs), 10 triacylglycerides (TAGs), 18 sphingomyelins (SMs), and 9 phosphatidylethanolamines (PEs). Meanwhile, 21 out of 215 common negative-mode features were assigned the same identity, 5 of which were PEs and 6 of which were phosphatidylinositols (PIs). Among the deuterium-labeled internal standards included by each laboratory, 6 were annotated and aligned across all four laboratory datasets in the positive mode, and 7 were this way in the negative mode. A total of 492 and 339 identified Table I features in the positive and negative modes were aligned to features in at least one other laboratory dataset; of these, 386 and 166 had matching identities in the other set(s). Most of the other aligned and annotated features were matched to unannotated compounds. Few mismatches were observed between the aligned features, with the most common stemming from negative-mode adducts labeled as [M+COOH]^−^ in dataset II vs. [M+CH_3_COO]^−^ in dataset I, thus differing by a single carbon. In the positive mode, there were 18 mismatches, mostly affecting annotations of PCs and PEs. As extensive validation of these annotations was not performed by each laboratory, metabCombiner alignments can serve as a secondary check to determine the concordance between feature annotations, as well as potential errors. 

### 3.2. Alignment of the Multi-Batch ELEMENT Study with batchCombine

**Figure 5:**
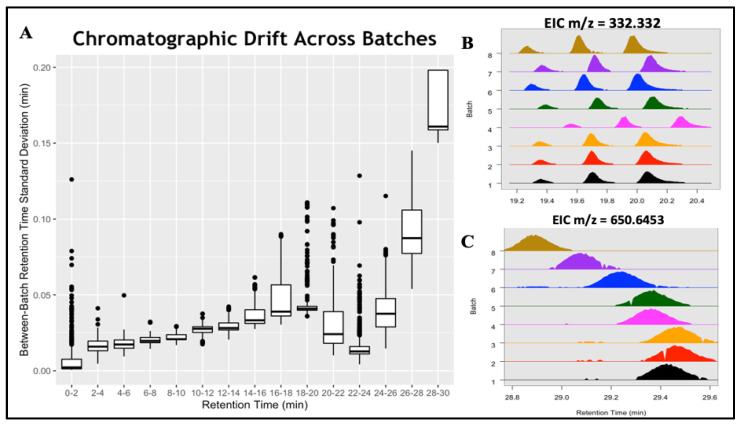
Inter-batch RT drifts in the ELEMENT study. (**A**) Boxplot of reported batch RT standard deviations across discrete chromatographic regions. (**B**) EIC of three similar-mass (m/z = 332.332) compounds, with a random shift observed in batch 4. (**C**) RT drifting of one compound (m/z = 650.6453) from batch 1 (29.4 min) to batch 8 (28.9 min).

We also demonstrate batchCombine on metabolomics data from ELEMENT, a well-characterized birth-cohort study that collected fasting serum from 402 Mexico City adolescents [13,19]. Samples were partitioned into eight batches, each composed of roughly fifty subject samples and nine pooled QCs. Raw LC-MS files were organized into their respective batches and separately pre-processed using XCMS [14], yielding 11,000 to 14,000 features per table. Each table was converted to metabData format, then aligned with batchCombine. The resulting batch-merged table contains 23,700 aligned feature rows in total; of these, 6493 were detected in all 8 batches, whereas 5453 were unique to one batch. Supplementary Sheet S2 illustrates a batch-aligned table output of batchCombine. The columns consist of reported feature meta-data for all eight experimental batches, including the m/z, RT, and Q values. These are useful for analyzing systematic and random drift in chromatographic and spectrometric measurements among the batches. Using the standard deviation of batch RTs ($\sigma_{rt}$) as a diagnostic, more than 75% of features found in two or more batches exhibited modest-to-no variation ($\sigma_{rt}$ < 0.03 min), whereas 5% to 10% of features exhibited more significant drifts between batches ($\sigma_{rt}$ > 0.05 min). The most significant variation was found near the end of the gradient (16–20 min) and the tail chromatographic region (26 min to 30 min), as illustrated in Figure 5A below. 

Two examples of aligned features that exhibited significant RT variation are depicted in Figure 5B,C. Figure 5B shows three isomers detected across all eight batches, with the measured m/z = 332.332 eluting between RT = 19.2 min and 20.3 min. A major chromatographic shift was observed for the three isomers in batch 4, with only minor variation detected in the remaining batches. In Figure 5C, a feature with m/z = 650.6453 exhibits gradual drifting from 29.5 min in the first three batches to 28.9 min in the last batch. Conventional alignment approaches perform poorly, with significant RT drifting exemplified here, assigning peaks arising from the same analyte as distinct entities. Errors like these often go undetected since most programs report a single m/z and RT value for each feature. Various computational multi-batch alignment tools perform post hoc alignment corrections of preprocessed XCMS data, using the presence or absence of peaks in at least one batch as a diagnostic criterion [6,8]. By contrast, the batchCombine approach is to perform within-batch alignments as a step towards between-batch LC-MS data merging, similar to the work of Liu et al. [7] This process is flexible and independent of pre-processing software, though it currently does not allow for “gap-filling” following alignment.

## 4. Discussion

metabCombiner 2.0 expands upon the original software while overcoming many of its limitations. Many additional improvements are being considered for the future development of this software. Though not observed in any of the studies presented here, systematic m/z drift has been reported to occur between datasets generated by different mass spectrometers [11]. Modules for visualizing and correcting drift across the range of measured m/z values will be implemented as a remedy for this issue. As illustrated in the inter-laboratory lipidomics study feature-alignment example, metabCombiner determines the overlaps of a chosen primary feature list with multiple secondary lists (all-to-one alignment), but leaves out intersections for features that are missing in the primary dataset (all-to-all alignment). The ability to incorporate features present in all datasets represents a major area of improvement for this tool. One potential solution is to perform an imputation procedure for combined dataset inputs, whereby quantitative descriptors (m/z, RT, Q) are projected onto a common reference. As this would potentially add thousands of entities to the feature list, multi-dataset alignment tasks are likely to grow in complexity. Steps to mitigate this, such as individual feature table post-processing (e.g., false-positive peak detection), should be implemented whenever possible. Finally, RT mapping between datasets remains the most challenging step towards the automation of the metabCombiner workflow due to numerous parameters (range constraints, anchor selection, and spline fitting) affecting the resulting fit. New functionality for optimization and diagnosing poor fits is a major priority for the future development of this software. 

## 5. Conclusions

This manuscript outlines upgrades to metabCombiner with the aim of extending nontraditional LC-MS alignment methods to combine multiple datasets or experimental batches. While the process is still based on a pairwise dataset-merging operation, the ability to perform the stepwise merging of multiple datasets represents a major improvement on the original capabilities of the software and opens new doors to exploring relationships between disparately acquired metabolomics data.

## Figures and Tables

**Table 1:** Inter-laboratory lipidomics study aligned-feature summary.

	Positive			Negative		
Detected Across Four Datasets	601			215		
Detected in I, II, and III	155			30		
Detected in I, II, and IV	280			115		
Detected in I, III, and IV	580			533		
Detected in I and II	156			211		
Detected in I and III	1053			398		
Detected in I and IV	743			670		
Detected in I only	5202			3691		
	II	III	IV	II	III	IV
Detected Outside I	2617	22,516	17,165	1676	6132	7402

## Data Availability

The raw LC-MS data for the unknown lipid study can be found in the Metabolomics Workbench with the dataset identifier ST002951. Links to the source code for the R package as well as the online version can be found at https://metabcombiner.medicine.umich.edu/ (accessed on 1 January 2024).

## Supplementary Materials

The following are available online at https://www.mdpi.com/article/10.3390/metabo14020125/s1, Text S1: Merging duplicate metabolomics features, Text S2: *metabCombiner* 2.0 script and guide, Text S3: batchCombine example script and guide, Table S1: One-to-one feature assignment with reduceTable, Table S2: Unknown lipids dataset feature counts, Table S3: Unknown lipids pairwise feature alignment counts, Figure S1: Pairwise retention time mapping curves, Sheet S1: Inter-laboratory lipidomics study alignment results, Sheet S2: ELEMENT study multi-batch alignment results.

## References

[1] Schrimpe-Rutledge A.C., Codreanu S.G., Sherrod S.D., McLean J.A. (2016). Untargeted Metabolomics Strategies—Challenges and Emerging Directions. J. Am. Soc. Mass. Spectrom..

[2] Smith R., Ventura D., Prince J.T. (2015). LC-MS Alignment in Theory and Practice: A Comprehensive Algorithmic Review. Brief. Bioinform..

[3] Boswell P.G., Schellenberg J.R., Carr P.W., Cohen J.D., Hegeman A.D. (2011). A Study on Retention “Projection” as a Supplementary Means for Compound Identification by Liquid Chromatography-Mass Spectrometry Capable of Predicting Retention with Different Gradients, Flow Rates, and Instruments. J. Chromatogr. A.

[4] Christin C., Smilde A.K., Hoefsloot H.C.J., Suits F., Bischoff R., Horvatovich P.L. (2008). Optimized Time Alignment Algorithm for LC−MS Data: Correlation Optimized Warping Using Component Detection Algorithm-Selected Mass Chromatograms. Anal. Chem..

[5] Abate-Pella D., Freund D.M., Ma Y., Simón-Manso Y., Hollender J., Broeckling C.D., Huhman D.V., Krokhin O.V., Stoll D.R., Hegeman A.D. (2015). Retention Projection Enables Accurate Calculation of Liquid Chromatographic Retention Times across Labs and Methods. J. Chromatogr. A.

[6] Brunius C., Shi L., Landberg R. (2016). Large-Scale Untargeted LC-MS Metabolomics Data Correction Using between-Batch Feature Alignment and Cluster-Based within-Batch Signal Intensity Drift Correction. Metabolomics.

[7] Liu Q., Walker D., Uppal K., Liu Z., Ma C., Tran V., Li S., Jones D.P., Yu T. (2020). Addressing the Batch Effect Issue for LC/MS Metabolomics Data in Data Preprocessing. Sci. Rep..

[8] Wu C.-T., Wang Y., Wang Y., Ebbels T., Karaman I., Graça G., Pinto R., Herrington D.M., Wang Y., Yu G. (2020). Targeted Realignment of LC-MS Profiles by Neighbor-Wise Compound-Specific Graphical Time Warping with Misalignment Detection. Bioinformatics.

[9] Hsu Y.-H.H., Churchhouse C., Pers T.H., Mercader J.M., Metspalu A., Fischer K., Fortney K., Morgen E.K., Gonzalez C., Gonzalez M.E. (2019). PAIRUP-MS: Pathway Analysis and Imputation to Relate Unknowns in Profiles from Mass Spectrometry-Based Metabolite Data. PLoS Comput. Biol..

[10] Mak T.D., Goudarzi M., Laiakis E.C., Stein S.E. (2020). Disparate Metabolomics Data Reassembler: A Novel Algorithm for Agglomerating Incongruent LC-MS Metabolomics Datasets. Anal. Chem..

[11] Climaco Pinto R., Karaman I., Lewis M.R., Hällqvist J., Kaluarachchi M., Graça G., Chekmeneva E., Durainayagam B., Ghanbari M., Ikram M.A. (2022). Finding Correspondence between Metabolomic Features in Untargeted Liquid Chromatography–Mass Spectrometry Metabolomics Datasets. Anal. Chem..

[12] Habra H., Kachman M., Bullock K., Clish C., Evans C.R., Karnovsky A. (2021). *metabCombiner*: Paired Untargeted LC-HRMS Metabolomics Feature Matching and Concatenation of Disparately Acquired Data Sets. Anal. Chem..

[13] Perng W., Tamayo-Ortiz M., Tang L., Sánchez B.N., Cantoral A., Meeker J.D., Dolinoy D.C., Roberts E.F., Martinez-Mier E.A., Lamadrid-Figueroa H. (2019). Early Life Exposure in Mexico to ENvironmental Toxicants (ELEMENT) Project. BMJ Open.

[14] Smith C.A., Want E.J., O’Maille G., Abagyan R., Siuzdak G. (2006). XCMS: Processing Mass Spectrometry Data for Metabolite Profiling Using Nonlinear Peak Alignment, Matching, and Identification. Anal. Chem..

[15] Pluskal T., Castillo S., Villar-Briones A., Orešič M. (2010). MZmine 2: Modular Framework for Processing, Visualizing, and Analyzing Mass Spectrometry-Based Molecular Profile Data. BMC Bioinform..

[16] Tsugawa H., Cajka T., Kind T., Ma Y., Higgins B., Ikeda K., Kanazawa M., VanderGheynst J., Fiehn O., Arita M. (2015). MS-DIAL: Data-Independent MS/MS Deconvolution for Comprehensive Metabolome Analysis. Nat. Methods.

[17] Shen T., Conway C., Rempfert K.R., Kyle J.E., Colby S.M., Gaul D.A., Habra H., Kong F., Bloodsworth K.J., Allen D. (2023). The Unknown Lipids Project: Harmonized Methods Improve Compound Identification and Data Reproducibility in an Inter-Laboratory Untargeted Lipidomics Study. biorXiv.

[18] Myers O.D., Sumner S.J., Li S., Barnes S., Du X. (2017). One Step Forward for Reducing False Positive and False Negative Compound Identifications from Mass Spectrometry Metabolomics Data: New Algorithms for Constructing Extracted Ion Chromatograms and Detecting Chromatographic Peaks. Anal. Chem..

[19] Rodríguez-Carmona Y., Meijer J.L., Zhou Y., Jansen E.C., Perng W., Banker M., Song P.X.K., Téllez-Rojo M.M., Cantoral A., Peterson K.E. (2022). Metabolomics Reveals Sex-specific Pathways Associated with Changes in Adiposity and Muscle Mass in a Cohort of Mexican Adolescents. Pediatr. Obes..

[20] Chambers M.C., Maclean B., Burke R., Amodei D., Ruderman D.L., Neumann S., Gatto L., Fischer B., Pratt B., Egertson J. (2012). A Cross-Platform Toolkit for Mass Spectrometry and Proteomics. Nat. Biotechnol..

[21] Tautenhahn R., Böttcher C., Neumann S. (2008). Highly Sensitive Feature Detection for High Resolution LC/MS. BMC Bioinform..

[22] Hohrenk L.L., Itzel F., Baetz N., Tuerk J., Vosough M., Schmidt T.C. (2020). Comparison of Software Tools for Liquid Chromatography–High-Resolution Mass Spectrometry Data Processing in Nontarget Screening of Environmental Samples. Anal. Chem..

[23] Clark T.N., Houriet J., Vidar W.S., Kellogg J.J., Todd D.A., Cech N.B., Linington R.G. (2021). Interlaboratory Comparison of Untargeted Mass Spectrometry Data Uncovers Underlying Causes for Variability. J. Nat. Prod..

[24] Liebisch G., Vizcaíno J.A., Köfeler H., Trötzmüller M., Griffiths W.J., Schmitz G., Spener F., Wakelam M.J.O. (2013). Shorthand Notation for Lipid Structures Derived from Mass Spectrometry. J. Lipid Res..

