# Supplementary material for: metabCombiner 2.0: Disparate Multi-Dataset Feature Alignment for LC-MS Metabolomics
Source: Metabolites. 2024 Feb 15;14(2):125. doi: 10.3390/metabo14020125 (PMC10891690; doi:10.3390/metabo14020125)
Supplement: Supplementary file 1 [file metabolites-14-00125-s001.zip › Supporting Information.pdf]

metabCombiner 2.0: Disparate Multi-dataset Feature Alignment for LC-MS Metabolomics

Hani Habra [1,*], Jennifer L. Meijer [2], Tong Shen [3], Oliver Fiehn [3], David A. Gaul [4], Facundo M. Fernández [4],Kaitlin R. Rempfert [5], Thomas O. Metz [5] , Karen E. Peterson [6,7], Charles R. Evans [8] and Alla Karnovsky [1]

1 Department of Computational Medicine and Bioinformatics, University of Michigan Medical School, Ann Arbor, MI 48109, USA; akarnovs@med.umich.edu
2 Department of Medicine, Geisel School of Medicine, Dartmouth College, Hanover, NH 03755, USA; jennifer.l.meijer@hitchcock.org
3 West Coast Metabolomics Center, University of California, Davis, CA 95616, USA; tsshen@ucdavis.edu (T.S.); ofiehn@ucdavis.edu (O.F.)
4 School of Chemistry and Biochemistry, Georgia Institute of Technology, 901 Atlantic Drive, Atlanta, GA 30332, USA; david.gaul@chemistry.gatech.edu (D.A.G.); facundo.fernandez@chemistry.gatech.edu (F.M.F.)
5 Biological Sciences Division, Pacific Northwest National Laboratory, Richland, WA 99352, USA; kaitlin.rempfert@pnnl.gov (K.R.R.); thomas.metz@pnnl.gov (T.O.M.)
6 Department of Nutritional Sciences, University of Michigan School of Public Health, Ann Arbor, MI 48109, USA; karenep@umich.edu
7 Department of Environmental Health Sciences, University of Michigan School of Public Health, Ann Arbor, MI 48109, USA
8 Department of Internal Medicine, University of Michigan Medical School, Ann Arbor, MI 48109, USA; chevans@med.umich.edu
* Correspondence: hhani@umich.edu

Contents

Supplementary Text S1: Merging Duplicate Metabolomics Features

Supplementary Text S2: *metabCombiner* 2.0 Script and Guide

Supplementary Text S3: *batchCombine* Example Script and Guide

Table S1: One-to-One Feature Assignment with *reduceTable*

Table S2: Unknown Lipids Data Set Feature Counts

Table S3: Unknown Lipids Pairwise Feature Alignment Counts

Figure S1: Pairwise Retention Time Mapping Curves

Supplementary Text S1. Merging Duplicate Metabolomics Features

Duplicate LC-MS features are two or more rows within very small m/z and RT differences describing the same compound. Duplicates are an artifact of many computational pre-processing methods and their presence has undesirable consequences for inter-dataset alignment. An example from the Unknown Lipids study is shown in the table below, with two separate rows (ΔRT = 0.01 min and Δm/z = 0.0029 Da) representing a single peak, one of which is annotated (17:0-16:1 PI-d5) and the other unannotated.

id	mz	rt	adduct	sample1	sample2	sample3	sample4	sample5
838.5535 @ 5.71	826.5523	5.47		748365	832033	761607	790608	755714
17:0-16:1 PI-d5	826.5494	5.46	[M-H]-	728640	791343	734634	795412	754102

When aligned to the other data sets, the two duplicate rows are matched to one corresponding feature each from tables III and IV, as determined by closest m/z. As in this scenario, feature duplication interferes with the accurate alignment of unique compounds, generating incomplete aligned rows.

id_I	id_III	id_IV	mz_I	mz_III	mz_IV	rt_I	rt_III	rt_IV	Q_I	Q_III	Q_IV
826.5494 @ 5.46	1_PI 17:0-16:1-d5		826.549	826.55		5.46	4.23		0.979	0.995	
17:0-16:1 PI-d5		17:0-16:1 PI-d5	826.552		826.552	5.47		4.37	0.978		0.99

While some preprocessing software packages have built-in functionality for resolving duplicate features, others leave unresolved duplicates in the data. *metabCombiner* provides two solutions for handling duplicates. The first is to retain a single copy with the lowest proportion of missingness or the highest median/ mean abundance, eliminating remaining rows. The main drawback of this approach is that information contained in the eliminated rows {e.g. named identifiers, adduct labels, or feature abundances} is lost. The alternative is to merge duplicates into a single representative feature row. Identifier and adduct labels are concatenated, and the highest abundance value per sample is retained. A weighted averaging option is available for recalculated, though superior alignment results were observed when using a single observed m/z and RT value.

Using the following command:

```
dataI <- metabData(tableI, samples = "sample", duplicate = opts.duplicate(mz = 0.005, rt = 0.05, "merge"))
```

merges the duplicate rows into a single merged row:

id	mz	rt	adduct	sample1	sample2	sample3	sample4	sample5
{838.5535 @ 5.71; 17:0-16:1 PI-d5}	826.549	5.47	[M-H]-	748365	832033	761607	795412	755714

Subsequently, in the results table, the aligned row is correctly assembled from features of the three tables:

id_I	id_III	id_IV	mz_I	mz_III	mz_IV	rt_I	rt_III	rt_IV	Q_I	Q_III	Q_IV
{826.5494 @ 5.46, 17:0-16:1 PI-d5}	1_PI 17:0-16:1-d5	17:0-16:1 PI-d5	826.549	826.55	826.552	5.47	4.23	4.37	0.98	0.995	0.99

Supplementary Text S2. *metabCombiner* 2.0 Script and Guide

This is a guide on using *metabCombiner* to align multiple data sets, using data sets from the Unknown Lipids Consortium study. All acquired data must meet the key method assumptions: untargeted metabolomics data of biologically similar specimens acquired with similar liquid chromatography methods in the same ionization mode, with no prior normalization which may distort the relative abundances of features (with respect to other features).

1) *metabCombiner* Installation

First, install *metabCombiner* from Bioconductor (release) or Github (development) and load the library. For this guide, we will install the release version; see the Github page for installing the development version.

```
#installing metabCombiner and loading packages necessary for demonstration
if (!require("BiocManager", quietly = TRUE))
    install.packages("BiocManager")
BiocManager::install("metabCombiner")
library(metabCombiner)
```

2) Loading Data and Initial Processing

The inputs to the program are pre-processed metabolomics feature tables generated from raw spectral files using open-source packages (XCMS, MZmine, MS-DIAL) or vendor software. Navigate to the directory where the files are located, load each one into R, and convert to metabData (single data set) format. Parameters 'mz', 'rt', 'id', 'adduct', 'samples' and 'extra' are customized keywords for mapping one or all columns whose names contain the indicated keyword(s). The remaining parameters are for the feature filters.

```
#reading in negative mode unknown lipids data files
setwd("path/to/directory")
lab_I <- read.csv("lab_I_UnknownLipids_InHouseMethod_NEG.csv")
data_I <- metabData(lab_I, samples = c("CHEAR", "NIST"),  rt = "RT", mz = "m/z", id = "Feature", rtmax = 14, rtmin = 0.3,
                    extra = names(lab_I), zero = TRUE, duplicate = opts.duplicate(mz = 0.0075, resolve = "merge"))
#It is possible to create metabData objects directly from the file name
data_II <- metabData("lab_II_UnknownLipids_InHouseMethod_NEG.csv ", mz = "Weight", rt = "RT",  id = "Name",
                    samples = "^Area: 0[0-3]_Plasma", id = "Name", extra = "^Area:",  zero = TRUE)
lab_III <- metabData("lab_III_UnknownLipids_InHouseMethod_NEG.csv ",  rt = "Rt", mz = "Mz",  id = "Metabolite",
                    samples = "Plasma[1-3]", adduct = "Adduct", extra = "NEG", zero = TRUE, misspc = 50))
lab_IV <- read,csv ("lab_IV_UnknownLipids_InHouseMethod_NEG.csv", rt = "adjusted row retention time", mz = "m/z",
                    id = "identity", samples = "Lip_Plasma", adduct = "Adduct", extra = "PNACIC", rtmin = 0.3, zero = TRUE)
```

3) First Pairwise Alignment

The first pair of data sets are aligned together, using the previously described *metabCombiner* workflow. We recommend providing unique identifiers to each data set in the *metabCombiner( )* step (xid and yid arguments). The choice of which data set to use as "xdata" or "ydara" is arbitrary, though we generally use the one with the longest chromatography time as "xdata" and the shorter one as "ydata".

```
# main package steps, as outlined previously
firstCycle <- metabCombiner(xdata = data_I, ydata = data_II, xid = "I",  yid = "II", binGap = 0.0075)
firstCycle <- selectAnchors(firstCycle, windx = 0.02, windy = 0.02, tolrtq = 0.2)
firstCycle <- fit_gam(firstCycle, k = 12, iterFilter = 1, rtx = c("min", 10), rty = c("min", 8.5))
plot(firstCycle, outlier = "s", xlab = "lab I", ylab = "lab II", main = "lab I vs lab II RT Mapping", lcol = "blue")
firstCycle <- calcScores(firstCycle, A = 60, B = 10, C = 0.2)
```

To complete the alignment, we reduce the aligned feature list using reduceTable( ). Afterwards, we use *updateTables*() to incorporate non-aligned features from the original xdata and ydata used to construct the object.

```
firstCycle <- reduceTable(firstCycle, maxRankX = 2, maxRankY = 2, maxRTerr = 0.5, delta = 0.2)
firstCycle <- updateTables(firstCycle, xdata = data_I, ydata = data_II)
```

Once complete, we have the first paired alignment of features in data sets I and II. This intermediate result serves as input for further alignment tasks.

4) Aligning Combined Dataset with Single Dataset

We can align the metabCombiner (combined data set) object of the previous steps with the next metabData (single data set) object. For the metabCombiner input, the data set identifier (xid for xdata, yid for ydata) of a representative data set can be specified among the existing identifiers; here, we select the data set with identifier *I*. A new identifier can be used to label the new single data set (e.g. *III*).

```
datasets(firstCycle)
# "I"     "II"

secondCycle <- metabCombiner(xdata = firstCycle, ydata = data_III,  xid = "I",  yid = "III", binGap = 0.0075)
```

The rest of the alignment process proceeds similarly to an alignment between a pair of single data sets.

```
# metabCombiner alignment steps: *I*-II vs III
secondCycle <- selectAnchors(secondCycle, windx = 0.02, windy = 0.02)
secondCycle <- fit_gam(secondCycle, k = 15, iterFilter = 1, rtx = c("min", 12.5))
plot(secondCycle, outlier = "s", xlab = "lab I", ylab = "lab III", main = "lab I vs lab III RT Mapping", lcol = "red")
secondCycle <- calcScores(secondCycle, A = 60, B = 12, C = 0.2)
secondCycle <- reduceTable(secondCycle, maxRankX = 2, maxRankY = 2, maxRTerr = 0.5, delta = 0.2)
secondCycle <- updateTables(secondCycle, xdata = firstCycle, ydata = data_III)
```

We repeat this process for yet another stepwise paired alignment, this time with a fourth data set (IV). For best results, use the same primary data set as before (e.g. I for this example) and for all subsequent alignment tasks. The *metabCombine()* wrapper function executes together the 6 function steps (*metabCombiner(), selectAnchors(), fit_gam(), calScores(), reduceTable(), updateTables()*). Using the plot method is still recommended for fine-tuning the RT mapping steps.

```
# metabCombiner alignment: *I*-II-III vs IV
thirdCycle <- metabCombine(xdata = secondCycle, ydata = data_IV,  xid = "I",  yid = "IV", binGap = 0.0075, union = TRUE,
                           anchorParam = selectAnchorsParam(tolmz = 0.002, tolrtq = 0.2),
                           fitParam = fitgamParam(k = 16, iterFilter = 1, rtx = c("min", 12)),
                           scoreParam  = calcScoresParam(A = 60, B = 12, C = 0.2),
                           labelParam = reduceTableParam(maxRTerr = 0.5, delta = 0.2))

plot(thirdCycle, outlier = "s", xlab = "lab I", ylab = "lab IV", main = "lab I vs lab IV RT Mapping", lcol = "purple")
```

5) Extracting and Writing Results

As described in the main text, there are two results tables generated as part of the metabCombiner alignment process: *combinedTable* and the *featData*. The *combinedTable* contains the results of the "active" or most recently performed pairwise alignment, including similarity scores, predicted RTs, and row labels. It contains all sample abundances and extra columns for each feature. The *featData* table contains metadata (id, m/z, RT, Q, adduct) for all aligned features across all the data sets. A separate method, *combineData()*, fuses elements of the two tables together to form a cohesive result (sans columns describing the active X and Y data set alignment).

```
#results tables
combined.table <- combinedTable(thirdCycle)
feature.data <- featData(thirdCycle)
combined.data <- combineData(thirdCycle)
```

Any of these tables can be written to file using the standard table writing R functions (e.g. write.table or write.csv).

```
write.csv(combined.data, file = "combined.dataset.csv", row.names = FALSE, na = "")
```

Supplementary Text S3. *batchCombine* Example Script and Guide

This is a brief guide on using *batchCombine* on the ELEMENT data set or similar large scale metabolomics studies composed of multiple experimental batches.

1) *metabCombiner* Installation

As before, *metabCombiner* must first be installed from Bioconductor (release) or Github (development) and loaded. The Bioconductor installation is provided in the below snippet.

```
#installing metabCombiner and loading packages necessary for demonstration
if (!require("BiocManager", quietly = TRUE))
    install.packages("BiocManager")
BiocManager::install("metabCombiner")
library(metabCombiner)
```

2) Formatting Directories and Files

batchCombine requires feature tables for each individual experimental batch, similar to the format described in Supplementary Text S2. It is highly recommended that the tables used for alignment have similarly named sample columns. In this example, pooled QC samples contain the label "CS0000009", and all other samples contain "S0003" in their names.

Navigate to the directory containing the raw .mzXML or .mzML files for the experiment. Organize the files into separate directories based on their respective batches (e.g. batch 1 raw files in a folder titled "Batch1"). Here, a directory called "RAW/POS" contains 8 subdirectories for each batch, each with their respective raw MS files.

```
#setting the working directory to the folder containing subdirectories for each batch of raw files
setwd("RAW/POS")

list.dirs()
#[1] "batch1" "batch2"  "batch3" "batch4" "batch5" "batch6"  "batch7" "batch8"

head(list.files("batch1"))
#[1] "20171017.EX00754.A003.IN0016.CS0000009.01.P"     "20171017.EX00754.A003.IN0016.CS0000009.02.P"
      "20171017.EX00754.A003.IN0016.CS0000009.03.P"     "20171017.EX00754.A003.IN0016.S00031217.P"
      "20171017.EX00754.A003.IN0016.S00031223.P"        "20171017.EX00754.A003.IN0016.S00031233.P"
```

Alternatively, pool all raw files into a single directory and use a metadata file containing the names of each raw experimental file organized by batch.

3) Pre-process with XCMS

[Note: If the batches are already pre-processed and outputted as separate files, skip to step #4]. batchCombine aligns batch feature tables pre-processed using any conventional software program. For simplicity, we use XCMS in this tutorial with the originally implemented functionality. See this link for detailed guides on using XCMS 3.0+

```
for(batch in list.files()){
      if(!dir.exists(batch))  next
      #XCMS workflow; parameters tailored to this data
      xset <- xcmsSet(batch, method = "centWave", peakwidth = c(3, 30),  noise  = 250, ppm = 20,
                              prefilter  = c(3,1000), snthresh  = 10, mzdiff  = -0.001, integrate  = 1,
                              fitgauss  = TRUE,  verbose.columns = FALSE)
      xset2 <- group(xset, method  = "density", bw  = 15, mzwid  = 0.015, minfrac = 0.5)
      xset3 <- retcor(xset2, smooth = "loess", family = "symmetric", missing = 5, span = 0.25)
      xset4 <- group(xset3, method  = "density", bw  = 5, mzwid  = 0.015, minfrac = 0.5)
      xset5 <- fillPeaks(xset4)
      res <- peakTable(xset5)
      res$rt <- res$rt / 60   #converting seconds to minutes
      write.csv(res, file = paste0(batch, ".csv"), row.names = FALSE, na = "")
}
```

The above code generates pre-processed tables for each of the batches, written as .csv files in the same directory.

4) Loading Data and Initial Processing

Each of the batch feature tables should be read into R as a list of data frames. We assume here that each of the tables are saved as .csv or .txt files.

```
#use .txt and read_delim if files are stored as .txt
filenames <- grep(".csv", list.files(), value = TRUE)
batches <- lapply(filenames, read.csv)
```

When using XCMS, no further formatting is necessary for use with *metabCombiner*, since all m/z columns are labeled as "mz", rt columns are "rt", and sample columns are similarly named. We convert this list of data frames into a list of formatted metabData objects.

```
batchData <- lapply(batches, metabData, mz = "mz", rt = "rt", zero = TRUE, id = NULL,
                    samples = "CS0000009", extra = "S000", misspc = 50)

#recommended step: give list elements unique identifiers, e.g. b1, b2, ....
names(batchData) <- paste("b", seq_along(batches), sep = "")
```

In the above example, pooled samples (columns containing CS0000009) are designated as central to quantitation comparisons, whereas all regular samples (containing S000) are brought into the results table as "extra" non-analyzed columns.

5) Setting Up *batchCombine* Parameters

Next, we set the parameters to be used for each pairwise combination. *batchCombine* will use default values for the various steps if these parameter values are not specified.

```
saparam <- selectAnchorsParam(tolmz = 0.003, tolQ = 0.3, tolrtq = 0.2, windx = 0.03, windy = 0.03)
fgparam <- fitgamParam(k = 20, iterFilter = 2)  #use only one value for k for faster execution
csparam <- calcScoresParam(A = 70, B = 35, C = 0.8)
rdparam <- reduceTableParam(minScore = 0.5, maxRTerr = 0.3)
```

6) Running *batchCombine* and Output

Finally, we proceed with the main step, which is the batchCombine function enveloping the list of metabData objects and the parameter lists. In this example, an m/z binGap value of 0.0075 is used for initial feature grouping, the union option is turned on for inclusion of non-intersected features, and all three quantitative descriptors (m/z, RT, Q) are averaged in each cycle using the means argument. The result is combinedRes, which contains a *metabCombiner* object organizing all feature information from each list as well as a merged feature table.

```
combinedRes <- batchCombine(batchData, binGap = 0.0075, union = TRUE,
                    anchorParam = saparam, fitParam = fgparam,
                    scoreParam = csparam, reduceParam = rdparam,
                    means = c(TRUE, TRUE, TRUE))
object <- combinedRes$object
results.table <- combinedRes$table
```

The object produced in this workflow can be used for additional alignments, treating each batch as a separate dataset. The results table can be written to a file and used for downstream analysis, e.g.

```
write.csv(results.table, file = "batch_merged_results.csv", row.names = FALSE, na = "")
```

Table S1. Unknown Lipids Data Set Feature Counts

Inter-laboratory Lipidomics Study Initial and Processed Feature Counts						
**Mode**	**Laboratory**	**Initial Count**	**RT Filter**	**Missingness Filter**	**Duplicate Feature Filter**	**Final Count**
**POS**	**I**	15004	-320	-3856	-2058	8770
	**II**	3819	0	0	-10	3809
	**III**	27289	0	-2278	-106	24905
	**IV**	29900	-17	-10508	-6	19369
**NEG**	**I**	10962	-1224	-2663	-1242	5863
	**II**	2262	0	0	-15	2247
	**III**	7643	0	-315	-20	7308
	**IV**	14865	-155	-5774	-1	8935

Table S2. Unknown Lipids Pairwise Feature Alignment Counts

	Positive		Negative	
**Data Set**	I	II	I	II
**Initial Feature Count**	15004	3819	10962	2262
**Filtered Feature Count**	8770	3809	5863	2247
**Grouped Features**	3350	2120	1828	899
**Final Feature Matches**	1192		571	

	Positive		Negative	
**Data Set**	I	III	I	III
**Initial Feature Count**	15004	27289	10962	7643
**Filtered Feature Count**	8770	24905	5863	7277
**Grouped Features**	6241	7939	3595	2298
**Final Feature Matches**	2389		1176	

	Positive		Negative	
**Data Set**	I	IV	I	IV
**Initial Feature Count**	15004	29900	10962	14865
**Filtered Feature Count**	8770	19369	5863	8935
**Grouped Features**	5711	5675	3755	2659
**Final Feature Matches**	2204		1533	

Table S2 These tables list the initial, filtered, m/z grouped, and intersected feature counts for each pairwise alignment between feature table I and tables (II, III, & IV) in the positive and negative ionization modes for the Unknown Lipids Consortium study.

Table S3. One-to-One Feature Assignment with *reduceTable*

A.

idx	idy	mzx	mzy	rtx	rty	rtProj	Qx	Qy	Score	rankX	rankY	label
BX	BY	265.1184	265.1182	4.781	5.979	5.9395	0.998	0.999	0.961	1	1	
BXX	BY	265.1161	265.1182	4.195	5.979	5.618	0.978	0.999	0.678	1	2	REMOVE

B.

idx	idy	mzx	mzy	rtx	rty	rtProj	Qx	Qy	score	rankX	rankY	label	resolveScore
BX	BYYY	209.0785	209.079	10.821	6.02	6.006	0.741	0.749	0.942	1	1	RESOLVED	0.942
BX	BYY	209.0785	209.079	10.821	5.76	6.006	0.741	0.5	0.806	2	1	REMOVE	0.806
BX	BY	209.0785	209.079	10.821	5.68	6.006	0.741	0.5335	0.792	3	1	REMOVE	0.792
BX	BYYYY	209.0785	209.079	10.821	6.16	6.006	0.741	0.1381	0.788	4	1	REMOVE	0.788

C.

idx	idy	mzx	mzy	rtx	rty	rtProj	Qx	Qy	score	rankX	rankY	label	resolveScore
CX	CYY	246.1706	246.1707	5.263	3.985	4.014	0.826	0.926	0.936	1	1	REMOVE	0.936
CXX	CYY	246.1707	246.1707	5.395	3.985	4.089	0.898	0.926	0.903	1	2	RESOLVED	1.798
CX	CY	246.1706	246.1706	5.263	3.911	4.014	0.826	0.884	0.895	2	1	RESOLVED	1.798
CXX	CY	246.1707	246.1706	5.395	3.911	4.089	0.898	0.884	0.84	2	2	REMOVE	0.84

D.

idx	idy	mzx	mzy	rtx	rty	rtProj	Qx	Qy	score	rankX	rankY	label
DX	DY	132.102	132.102	2.354	1.502	1.51	0.999	0.998	0.969	1	1	RESOLVED
DXX	DYY	132.102	132.102	2.54	1.634	1.591	1.000	1.000	0.955	1	1	RESOLVED
DXX	DY	132.102	132.102	2.54	1.502	1.591	1.000	0.998	0.94	2	2	REMOVE
DX	DYY	132.102	132.102	2.354	1.634	1.51	0.999	1.000	0.93	2	2	REMOVE
DXXX	DYY	132.102	132.102	8.108	1.634	5.036	0.74	1.000	0.255	1	3	REMOVE
DXXX	DY	132.102	132.102	8.108	1.502	5.036	0.74	0.998	0.128	2	3	REMOVE
DXXX	DYYY	132.102	132.102	8.108	10.155	5.036	0.74	0.206	0.035	3	1	REMOVE
DXX	DYYY	132.102	132.102	2.54	10.155	1.591	1.000	0.206	0.035	3	2	REMOVE
DX	DYYY	132.102	132.102	2.354	10.155	1.51	1.000	0.206	0.034	3	3	REMOVE

Table S3 These examples illustrate how *metabCombiner* assigns one-to-one correspondence between feature pairs in the reduceTable( ) function. For all cases, the minimum score threshold (minScore) is 0.5, the rank thresholds (maxRankX and maxRankY) are 3, and delta score (delta) is 0.2. In addition, the consistent retention time order (rtOrder) condition is expected for conflict resolution. In **(A)**, two candidate X features are aligned to a single Y feature, but the first pair AX - AY has alignment score that's over 0.23 higher than the second candidate pair, therefore the lower pair is eliminated. In **(B)**, a single X compound is matched to four possible Y compounds, all meeting the threshold parameters. In this scenario, only the top row is retained as only a single row can be selected for compound BX. In **(C)**, an isomer pair (CX and CXX) is mapped to its counterparts (CY and CYY), with very competitive scores. Although the alignment of CX with CYY scores the highest, the row combination (CXX - CYY & CX - CY) achieves one-to-one correspondence with the highest sum of scores (1.798) and preserves RT order, whereas (CX-CYY & CXX-CY) fails the RT order condition. **(D)** The score threshold eliminates the five lower rows, leaving compounds DXXX and DYYY without any possible matches. As for the top four rows, both DXX-DY and DX-DYY conflict with multiple top-scoring alignments (DX-DY and DXX-DYY), which automatically renders them as removable rows.

Figure S1. Pairwise Retention Time Mapping Curves

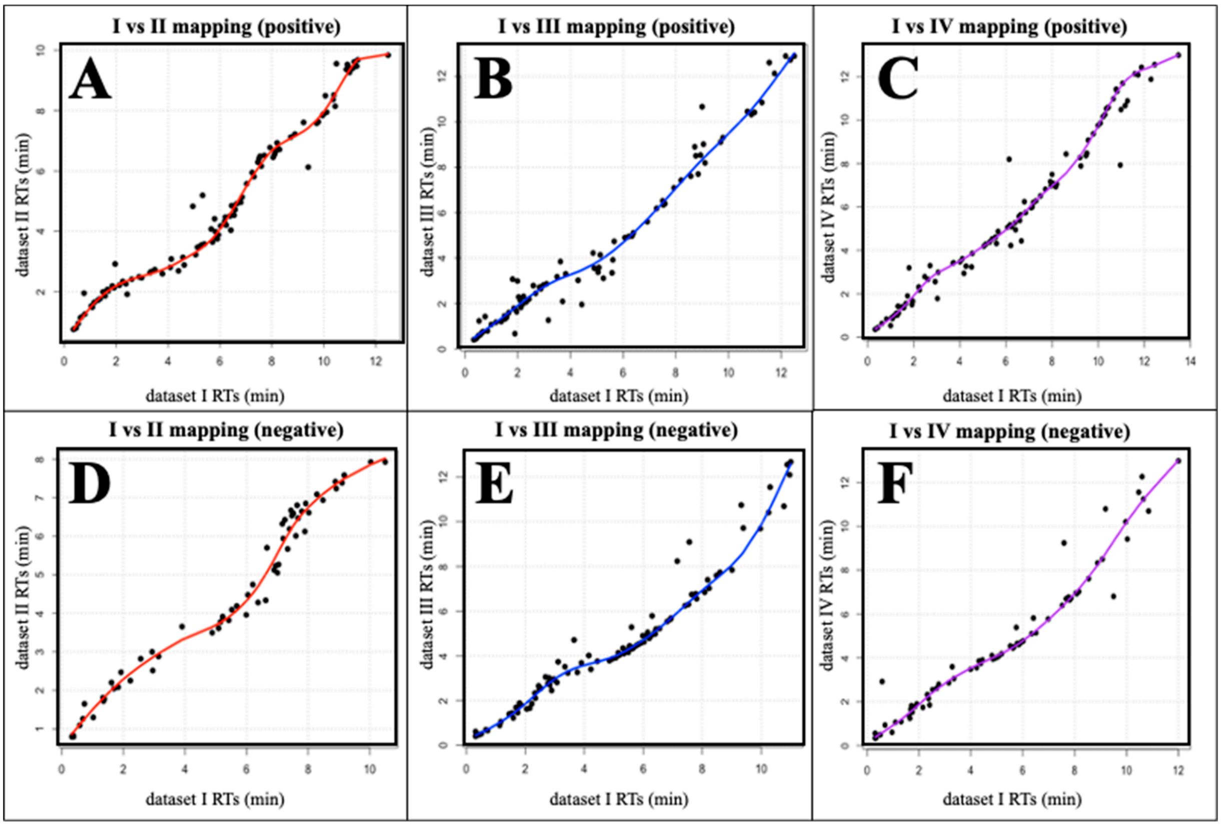

Figure S1 (A-F) Pairwise RT mapping curves in the positive and negative ionization modes from the Unknown Lipids data sets. (A-C) depict the positive mode data set alignments; (D-F) illustrate negative mode alignments. In each process, the chromatographic RTs of data set I are projected onto those of data sets II, III, or IV for comparison in similarity score calculations. RT endpoints (rt_{min} and rt_{max}) are adjusted in each alignment case, according to how well the chosen anchors span the chromatographic range.
